# An Account of a Method of Performing the Operation of Lithotomy at Two Different Times

**Published:** 1789

**Authors:** Petrus Camper

**Affiliations:** Honorary Professor of Physic, Anatomy, and Surgery at Amsterdam, Fellow of the Royal College of Physicians and of the Royal Society of Edinburgh, of the Imperial Acadamy at St. Petersburgh, and of the Royal Academy of Sciences and Royal Medical Society at Paris, &c.


### VI. *An Account of a Method of performing the Operation of Lithotomy at two different Times. By* Petrus Camper, *M. D. F. R. S. Honorary Professor of Physic, Anatomy, and Surgery at Amsterdam, Fellow of the Royal College of Physicians and of the Royal Society of Edinburgh, of the Imperial Academy at St. Petersburgh, and of the Royal Academy of Sciences and Royal Medical Society at Paris,* &c. *Translated* from the Dutch.*

I SHALL begin this account of an improved method of performing the operation of lithotomy, which the celebrated M. Louis has for some time adopted with great success, by mentioning the information I was favoured with lately, when at Paris, by M. Louis himself on this subject.

* Just as this article (which is extracted from the *Nieuwe Vaderlandsche Letteroeffeningen*, a periodical work published at Amsterdam) was going to the press, we received, with infinite regret, an account of the death of the learned and benevolent Author, at the Hague, (where he was a Member of the Council of State) in his 67th year, April 7th, 1789.— EDITOR.

His

His uſual method, as he obſerved to me, of cutting for the ſtone, is by the lateral operation as improved by Hawkins, and which is too well known to render any account of it neceſſary on the preſent occaſion. M. Louis is of opinion that, in this operation, it is not the inciſion into the bladder which occaſions the greateſt difficulty in the cure, but rather the immediate removal of the ſtone as ſoon as the inciſion is made; and this for theſe obvious reaſons, that the bladder muſt neceſſarily be in a painful and irritable ſtate from the introduction of the ſtaff, and liable to be injured by the exertions that may be requiſite to extract the ſtone immediately. In caſes of this ſort, he obſerves, the bladder, until the inciſion is made, ſuffers violent pain from the difficulty with which the urine is voided, and likewiſe from the introduction of the ſtaff and other inſtruments requiſite in the operation; and the conſequence of this is a conſtant and powerful contraction of the irritated part, ſo that one can only, as it were, by force get into the bladder to take hold of and extract the ſtone. Indeed I myſelf can affirm that both during the extraction of the ſtone, and afterwards, I have frequently ſeen very alarming ſymptoms take place, although the operation was performed by very able and experienced furgeons.

M. Louis affured me, that fince he had omitted to extract the ftone immediately after the incifion he had not loft a fingle patient by lithotomy, an operation which is in general, with good reafon, confidered as a very dangerous one*. On the day appointed for the operation, his practice for fome time paft has been to proceed no farther in it than the making the incifion into the bladder †. As foon as this part of the ope-

* It has been well obferved by one of the lateft and moft judicious Writers on Surgery, that the hazard attending the operation of lithotomy may be confidered as correfponding with the fize of the ftone to be extracted. In healthy fubjects, when the ftone is fmall, and the operation is properly performed, he is inclined to think that not above one dies in twenty. But although a few inftances have occurred of patients recovering from whom ftones have been extracted of a large fize, yet, whenever the ftone exceeds feven or eight ounces in weight, he is of opinion that not above one in ten recovers.—See Mr. Bell's Syftem of Surgery, Vol. II. page 114.——EDITOR.

† Although M. Louis has adopted Hawkins's method, yet he thinks the other modes of performing the lateral operation, as practifed by Rau, Chefelden, and Le Dran, may be quite as advantageous as that is when the operator is accuftomed to them.

ration ration is accompliſhed, the patient, who, by means of the opening made into the bladder, has a free and eaſy outlet for his urine, is put to bed, and the next day M. Louis examines the wound carefully, and endeavours to aſcertain what nature has done, or is actually doing, to rid herſelf of the ſtone. He ſometimes waits till the third, fourth, or even fifth day after the inciſion before he attempts to extract the ſtone by means of an inſtrument, and the removal of it then, he obſerves, is ſo eaſy to the operator, and ſo little painful to the patient, as would *a priori* be deemed almoſt incredible.

In caſe there are ſeveral calculi, he proceeds in the ſame cautious manner, and waits for a favourable opportunity to extract them.

The great advantages of this method ſtruck me very forcibly, and ſeemed eaſy of explanation from analogy with thoſe caſes in which we ſee other extraneous bodies, ſuch as glaſs, bullets, portions of fractured bone, &c., make their way gradually outwards by ſuppuration, and as it were almoſt inſenſibly, by the powers of nature alone; and I can eaſily conceive that the ſtone, as an extraneous ſubſtance, will, after the inciſion into the bladder, procure itſelf an exit in the ſame manner. Indeed I am able to recollect recollect inftances where, after the lateral operation, ftones left behind in the bladder have been difcharged through the wound almoft without the knowledge of the patient: I had, therefore, no doubt of the good effects of this mode of treatment.

This improvement is not fo new as might perhaps be imagined; for M. Louis has traced it to the time of Peter Franco, who, more than two centuries ago, recommended the fame method of operating.

I had no time while at Paris to look into Franco's work; but fince my return I have perufed a copy of this fcarce book, for which I am indebted to Profeffor Roell. I call it a fcarce book, becaufe there is no copy of it in the libraries belonging to the Univerfities of Leyden, Franeker, or Groningen. It is not in the collection publifhed by Uffenbach *; and even Haller, who feems to have been acquainted with the general merit of Franco as a furgical writer, has paid but little attention † to the particular paffages here alluded to.

My

* Thefaurus Chirurgicus. Folio. Francof. 1590.

† *Effe ubi præftet lapillum poft veficam incifam fibi relinquere, qui facilius fub tempore fuppurationis elabatur,* is all that

My copy of Franco's book * is dated Lyons, 1561; but, according to Haller, a former edition of it had appeared ſome years before, viz. in 1556, under another title.

In this work he treats very fully of different ſurgical methods of treatment, and in particular of lithotomy.

In his thirty-ſecond chapter, ſpeaking of the fatigue a patient undergoes when there is more than one ſtone in the bladder, he makes uſe of the following expreſſions: — "Some," ſays he, "have kept their patients ſo long under their "hands, that they have died. It would be "better, therefore, to do it at two times, as will "hereafter be ſhown, than thus to haſten death.

that Haller ſays of this method in his account of Franco's book.— See his Bibl. Chirurg. Tom. I. p. 211.— EDITOR.

* A copy of it is in the Britiſh Muſeum. It is entitled "Traité des Hernies; contenant une ample declaration de "toutes leurs eſpeces, et autres excellentes parties de la Chi-"rurgie, aſſauoir de la Pierre, des Cataractes des yeux et "autres maladies, deſquelles comme la cure eſt perilleuſe, "auſſi eſt elle de peu d'hommes bien exercée: avec leurs "cauſes, ſignes, accidens, anatomie des parties affectées, et "de leur entiere gueriſon. Par *Pierre Franco* de Turiers "en Provence, demeurant a preſent a Orenge." 8vo. Lyons, 1561. — EDITOR.

4 " . . . . Never-

"....Neverthelefs, as the wound is open, the
"urine, for fome days, flows out more eafily,
"and without fo much pain; though it is true
"that when the ftone comes to lay upon the
"wound this cannot be done without pain.
"When, therefore, we find that another ftone
"is remaining, we muft try to bring it away,
"if the patient is free from fever, and there is
"nothing elfe to prevent it; for moft frequent-
"ly they prefent themfelves fpontaneoufly at
"the wound, whether there be one or feveral
"ftones. It is then eafy to draw them out
"through the wound; but in cafe they fhould
"not come down and prefent of themfelves,
"we muft then make ufe of the method I have
"before defcribed for extracting them *."

In another place he fays, "If the ftone is
"large or uneven, the ftrength of the patient
"may

* "Aucuns ont tant tenu les patiens en leurs mains, qu'ils

"font demeurez morts. Il vaudroit mieux le faire a deux

"fois, comme fera cy après monftré, que de les precipiter a

"la mort.... Toutes fois a caufe que la playe eft ouverte,

"l'urine paffe plus facilement par quelques jours fans tant de

"douleur; bien eft vray que quand la pierre fe vient appuyer

"fur l'ulcere, que ce ne fe peult faire fans douleur. Ayant

"donc entendu et connu qu'il y peult encores avoir pierre,

"il fault effayer la tirer fi le patient eft exempte de fievre, et

"autre

"may be exhaufted, or he may even die under "the hands of the furgeon, and this as much "from the pain as from the great lofs of blood. ". . . . I find it better, therefore, (as I have "done feveral times) to do it at two times *."

Franco defcribes this mode of operating in the following words:—"Firft," fays he, "the "patient muft be made ready, and after- "wards the incifion is to be made in the "fame manner neither more nor lefs than was "directed in the preceding chapter: this be- "ing done, a tent, if you will, may be intro- "duced, there being no occafion at this time "to make any attempt to get away the ftone, "unlefs it fhould chance to prefent itfelf at the "wound; but, after making the incifion, dref-

"autre chofe n'empeche, car le plus fouvent elles fe viennent "rendre d'elles mefmes à la playe, foit qu'il en y ait une ou "plus. Alors eft facile les tirer hors par la playe mefme. "Et fi d'elles mefmes ne defcendoyent bas, et que ne fe pre- "fentaffent, il fault ufer des moyens que avons dit cy devant "pour les y amener."—Franco, Traité des Hernies, p. 128.

* "Si la pierre eft groffe ou roigneufe, la force du patient "peut eftre profternée, ou bien demeurer entre les mains du "maiftre, tant a raifon de la douleur, que de la grande fluxion "du fang. . . . Le trouve donc meiller (comme j'ay fait plu- "fieurs fois) de le faire en deux fois."—Ibid. p. 123.

" fings muft be applied to the wound, with ban-

" dages, as before mentioned. After fome

" days, when the patient appears to be in a

" good ftate, and without fever, (which is not

" to be expected unlefs he keeps to a good

" regimen) if the ftone prefents itfelf at the

" wound, which it moft frequently does, as I

" have feveral times experienced, it muft then

" be extracted in the manner already defcribed.

" But if it fhould happen not to prefent itfelf,

" it muft be brought down by introducing the

" finger into the rectum, and compreffing the

" lower part of the belly *."

* " Premierement il fault que le patient foit preparé, et

" apres faire l'incifion en la mefme facon ne plus ne moins

" qu'avons dit au chapitre precedent: et l'ayant faite on

" pourra mettre une tente fi l'on veult; n'eftant befoin de rien

" tenter apres la pierre pour cefte fois, fi d'aventure ne fe pre-

" entoit d'elle mefme à la playe: apres avoir faite l'incifion,

" fault mettre les appareils deffus la playe avec bendages,

" comme deffus. Apres quelques jours quand on connoiftra

" le patient eftre en bonne difpofition et fans fievre (laquelle

" ne luy adviendra moyennant qu'il tienne bon regime) fi la

" pierre fe prefentoit a la playe, comme le plus fouvent fait,

" ainfi qu'ay par plufieurs fois experimenté, faudra la tirer

" fuyvant la maniere expofée. Mais ne fe prefentant point il

" a fault faire defcendre en mettant les doigts au fondement,

" et en comprimant le petit ventre."—Ibid. p. 134.

He

He ſpeaks of this as a method peculiar to himſelf, and not deſcribed by any former writer.—"Some," he obſerves, "may think it "ſtrange to leave a patient in this manner, "undiſturbed, the ſpace of five or ſix days, "more or leſs, after making the inciſion. It "is certain, however, that perſons of good "judgment, when they have heard the rea- "ſons, have been ſatisfied *."

He farther confirms the utility of this mode of treatment by the following remarks:—"It has "ſometimes happened," ſays he, "that, after "having extracted a ſtone, the patient has been "ſo weak I have not dared to undertake any "farther examination to know whether any "other remained, fearing leſt he ſhould die "under my hands. But having put the dreſ- "ſings on the wound, and bound up the pa- "tient in the manner already deſcribed, I have "left him till he was become ſtronger, and "very often have found, in changing the firſt "dreſſings, or afterwards, that the ſtone which

* "Aucuns le trouvent eſtrange de laiſſer ſon patient ainſi "en repos l'eſpace de cinq ou ſix jours plus ou moins apres "avoir fait l'inciſion. Bien eſt vray que gens de bon juge- "ment, quand ils ont entendu les raiſons ont eſté ſatisfaits." —Ibid. p. 138.

"had remained was come entirely away of it-
"felf.... Obferving thefe things, and having
"feveral times experienced them, I have fal-
"len on the method contained in this chapter;
"that is to fay, that, after the incifion is made,
"the ftone is not to be immediately extracted
"unlefs it prefents of itfelf*."

The foregoing paffages will be fufficient to fhow the accurate and judicious manner in which Franco has treated this fubject, and how well he deferves to be quoted as an authority for this mode of treatment by my experienced friend, M. Louis. The latter, however, deviates a little from Franco's method, by leaving a free paffage for the urine inftead of filling up

* "Meftant quelque fois advenu, que apres avoir tiré une
"pierre le patient eftoit tant debile, que je n'aufoye entre-
"prendre de le plus preffer, pour favoir s'il y en demeuroit
"point d'autre, creignant qu'il ne mouruft entre mes mains.
"Or ayant mis les appareils fur la playe, et bendé comme
"avons dit deffus, je le laiffoye jufques a ce qu'il fuft plus
"fort, et bien fouvent ay trouvé que en changeant le premier
"appareil, ou appreft, que la pierre qui eftoit demeurée ef-
"toit fortie du tout dehors d'elle mefme....Voyant ces
"chofes, et les ayant par plufieurs fois pratiquées, j'ay col-
"ligé cette methode, contenue en ce chapitre: affavoir qu'a-
"pres l'incifion faite de ne tirer la pierre tout a la fois fi
"d'elle mefme ne s'y prefentoit."—Ibid. p. 138 & 139.

the wound, and this he does, with a view to prevent unneceſſary pain to the patient.

It will perhaps be a ſtill greater recommendation of this method when I add, that it is highly extolled by Fabricius Hildanus in his excellent book * on lithotomy. Manget alſo, in his *Bibliotheca Chirurgica* †, approves of this method.

* In his 16th chapter, entitled " De quarto operandi mo-" do, in Lithotomia uſitato, qui meritó *Lithotomia Franco-" nina* appellari poteſt," in which, after treating fully of the method of operating deſcribed in the preſent paper, he adds his own teſtimony in its favour in the following words:— " Quam probus ac prudens Lithotomus Francus iſte fuerit, " apparet. Idem quoque familiares ipſius de ipſo teſtati ſunt, " quorum adhuc aliqui, cum anno 1586 Lauſannam * ve-" niſſem, in vivis ſuperſtites fuerant. Utinam operandi mo-" dus iſte apud omnes lithotomos et caſtratores introduci poſ-" ſet; longé enim plures inciſos evaſuros exiſtimo. Quoniam " enim lithotomus ita apud ſe ſtatuit, neceſſe eſſe, ut æger " prima vice vel a calculo liberetur, aut moriatur, hinc ſæpe " fit, ut in inciſione tantam profundat ſanguinis copiam, vel " adeo torqueatur, ut propter dolorem immanem, inflamma-" tionem, aliaque perniciosa ſymptomata mox ſubſequentia, " vel in ipſa inciſione vel ſtatim poſt moriatur. Quæ omnia " impediri poſſent, ſi præſcriptum operandi modum imitare-" mur."——EDITOR.

† Tom. I. p. 274.

* Franco reſided many years at Lauſanne.

I muſt

I muſt not omit to mention that Colot ſometimes performed the operation of cutting for the ſtone at two different times. In his work * on lithotomy he obſerves † that this method made much noiſe at Court, and that the King had expreſſed himſelf pleaſed with the invention. Colot makes no mention of Franco in this part of his book, and indeed he adviſes this method only in caſes of great debility; for in general he directs ‡ the ſtone to be extracted immediately after the inciſion, leſt the ſurgeon ſhould find himſelf obliged to perform this part

* Traité de l'Operation de la Taille; avec des obſervations ſur la formation de la pierre et les ſuppreſſions d'Urine. Ouvrage poſthume de M. *Fr. Colot*; auquel on a joint un diſcours ſur la methode de Franco et ſur celle de M. Rau. 8vo. Paris, 1727.

† "Ce fut la cure de M. l'Abbé de Chauvelin qui me "donna lieu de faire l'operation de la pierre en deux tems; "cela fit beaucoup de bruit, particulierement a la cour, où "ſa Majeſté loua cette decouverte."—Colot, p. 182.

‡ "Pour ceux qui ſont dans les grands accés de leurs dou"leurs, il faut enlever la pierre des qu'on a fait l'ouverture; "autrement on riſque de ſe voir obligé de tirer la pierre dans "le tems ou la nature travaille a faire la ſuppuration de la "playe, ce qui l'empecheroit de pourſuivre ſon chemin . . . . "Il eſt donc tres rare de pouvoir obtenir ce que l'on ſe pro"poſe de cette methode."—Ibid. p. 183.

of of the operation when the wound has begun to uppurate: and he adds, that the advantages the ſurgeon may propoſe to himſelf from this method will be very ſeldom obtained.

In caſes where the patient's ſtrength is extremely reduced, Heiſter * adviſes Colot's method to be adopted; as he does likewiſe when there are ſeveral calculi in the bladder: and we are told by Saviard, that, in the caſe of a child who had two calculi, finding the patient greatly fatigued after the removal of the firſt, he ſuffered eight days to elapſe before he proceeded to the extraction of the remaining calculus, and the patient, he adds, was perfectly cured †. Theſe authorities all tend to confirm the practi-

* Syſtem of Surgery, Part II. ſect. v. chap. 141.

† "Je pouſſay enſuite pour la ſeconde fois mon bouton "dans la veſſie, où je ſentis une ſeconde pierre, que je ne ju"geay pas à propos de tirer ce jour là parceque l'enfant etoit "trop fatigué. Je le fis panſer avec les aſtringens, les em"brocations, et tous les remedes dont on ſe ſert au premier ap"pareil; on luy fit le lendemain une petite ſaignée, et l'on "continua pendant huit jours à le panſer, avant que je ſon"geaſſe a lui tirer cette ſeconde pierre, que je chargeay et "tiray enſuite comme la premiere. . . .Le panſement fut con"tinué, avec beaucoup d'exactitude, juſqu'a ſa parfaite gue"riſon."— *Vide* Nouveau Recueil d'Obſervations Chirurgicales. 8vo. Paris, 1702, p. 206.

cability cability and good effects, in certain cafes, of Franco's method.

The great object of M. Louis, that of leffening, by means of the incifion, the violent pain the patients experience, may be fupported by the authority of feveral eminent writers on furgery, although they have in general recommended it only when the ftone is too large to be extracted. Thus Baron Van Swieten, in his Commentary on the 1427th aphorifm of Boerhaave, fpeaking of the occurrence of a ftone in the bladder too large to be extracted, obferves, that in fuch cafes nothing more remains to be done than to make an artificial opening in the perinæum, through which the urine may be evacuated. By this means, he adds, "the evil "may be palliated, though not removed, and "life will be rendered more tolerable *." The learned Commentator adds, that this method has been tried with a good effect by Douglas, on the authority of *Collet*, a famous French lithotomift. I have no doubt but that for *Collet* we fhould read *Colot*, as the obfervation mentioned by Van Swieten, apparently on the au-

* "Leniuntur fic mala, licet non tollantur, redditurque "vita tolerabilior."

thority thority of Douglas, is to be found in Colot's work * already referred to.

It appears, however, from a paſſage in Avicenna, that an opening into the urethra, for the ſake of letting off the urine in caſes of pain from the ſtone, was in uſe many centuries ago. The paſſage in queſtion, as I find it in a Latin verſion † of Avicenna, may be thus rendered: "When there is difficulty in diſcharging the "urine, occaſioned by a ſtone in the bladder, "and there is no poſſibility of extracting the "ſtone by cutting, on account of ſome parti-"cular circumſtance that prevents it, or the "fears of the patient; in ſuch caſes a ſmall "opening muſt be made in that part which is "between the anus and teſticles, and into this "opening a canula introduced, that ſo death "may be prevented, although life will thereby "not be rendered thoroughly eaſy."

