# Errata

**Published:** 1789

**Authors:** 


					ERRATA.
page 175, for itpfuraie, read fuppuratc.

				

